# Disparities in Low‐Birth‐Weight Prevalence Across Kenya: A Systematic Review and Meta‐Analysis of Maternal, Demographic, and Socioeconomic Risk Factors

**DOI:** 10.1002/hsr2.72404

**Published:** 2026-04-19

**Authors:** Wusa Makena, Monday Nwankwo, Aisha Idris, Onyinoyi Bethel Onimisi, Moses Sunday Adefisayo, Patrick Maduabuchi Aja, Ibe Michael Usman, Elna Owembabazi, Elizabeth Bassy Umoren, Ilemobayo Victor Fasogbon, Onyinye Vivian Ojiakor, Chinyere Nkemjika Anyanwu

**Affiliations:** ^1^ Department of Human Anatomy, Faculty of Biomedical Sciences Kampala International University, Western Campus Bushenyi Western Region Uganda; ^2^ Department of Human Anatomy, Faculty of Basic Medical Sciences Federal University Lafia Lafia Nigeria; ^3^ Department of Human Physiology, Faculty of Basic Medical Sciences, College of Medicine Kaduna State University Kaduna Nigeria; ^4^ Department of Human Anatomy Usmanu Danfodiyo University Sokoto Sokoto State Nigeria; ^5^ World Health Organization United Nations House Abuja Nigeria; ^6^ Department of Biochemistry, Faculty of Biomedical Sciences Kampala International University, Western Campus Bushenyi Uganda; ^7^ Department of Physiology, Faculty of Biomedical Sciences Kampala International University, Western Campus Bushenyi Uganda; ^8^ Department of Microbiology, Faculty of Biomedical Sciences Kampala International University, Western Campus Bushenyi Uganda

**Keywords:** Kenya, low birth weight, meta‐analysis, prevalence, sociodemographic

## Abstract

**Background and Aims:**

Low birth weight (LBW) persistently poses a substantial public health risk throughout Kenya, most intensely within lower‐middle‐income groups. The research aimed at determining the prevalence of LBW in Kenya while conducting an evaluation of maternal and sociodemographic factors related to its development. A systematic review of observational studies (cross‐sectional and cohort) was conducted to assess LBW prevalence together with risk factors.

**Methods:**

The research utilized multiple databases to run a thorough literature search in accordance with PRISMA guidelines from 2000 up to November 2024. The research included sixteen studies that satisfied the established criteria. A random‐effects model was utilized to evaluate both LBW prevalence as well as risk factor prevalence levels. The statistical approach for assessing heterogeneity consisted of both Cochran's Q‐test and the I² statistic. The study analyzed variation by using subgroup assessments together with meta‐regression evaluation methods.

**Results:**

Research findings indicated a combined prevalence of 11.7% (95% CI: 8.9%–15.1%) for LBW in Kenya alongside significant variability (I² = 98%). Mothers without formal education demonstrated the peak rate of 13.2% LBW, while mothers with tertiary education showed the lowest LBW rate at 8.5%. Rates of Low birth weight demonstrated a connection with birth order position since sixth or higher birth order infants, along with first‐born infants, presented greater LBW risk (8.6% and 8.1%) than fourth or fifth order delivery (5.9%).

**Conclusions:**

This systematic review and meta‐analysis confirm that LBW, with a prevalence of 11.7% in Kenya, remains a major issue that varies by region and maternal factors. The most at‐risk are uneducated mothers and firstborns of high birth order. There should be region‐specific policies to improve maternal health services and quality prenatal care. Additionally, promoting female education is crucial to reducing LBW and its long‐term impacts.

## 1 Introduction

The World Health Organization (WHO) defines Low birth weight (LBW) as a birth weight that falls below 2500 grams (5.5 pounds), thus making this condition a major worldwide public health issue. The condition creates significant health risks for newborns by putting them at increased risk of death during the neonatal period and advancing the development of both developmental delays and long‐term chronic diseases [1].

The prevalence of LBW persists as a major health issue because 19.8 million newborns among total live births were classified as LBW during 2020. Research shows that the highest rates of LBW exist in Southern Asia and sub‐Saharan Africa, because these regions make up greater than 70% of all cases [2, 3]. The Global Nutrition Target sets a goal of reducing LBW by 30%, but current progress does not match the required pace [2].

The medical research connects LBW to neonatal fatality rates as well as the establishment of lasting healthcare difficulties, which combine growth issues with cognitive impairment and higher odds of developing health conditions like hypertension and diabetes in future life [3]. Physical development and mental advancement of children depend critically on birth weight, which influences their future health progress throughout life [3].

Medical studies prove that most LBW‐related neonatal fatalities originate from infections, including neonatal sepsis, resulting in 21.9% infant mortality rates [4]. Research studies done in East Java have proven that greater rates of LBW directly cause higher neonatal death rates while demonstrating significant statistical associations through *p*‐values [5].

LBW occurrence between pregnant women depends on multiple maternal elements that include maternal age as well as hypertension, diabetes, anemia status, and insufficient prenatal medical care [6]. The birth weight outcomes are influenced by three key determinants, which include fetal sex, maternal pregnancy history as represented by the number of pregnancies, and inter‐pregnancy interval [6]. Socioeconomic and environmental elements jointly create essential conditions with biological factors because they determine maternal accessibility to healthcare and nutrition levels [7].

Maternal age stands as an essential risk factor influencing LBW delivery rates because mothers under twenty years or above thirty‐5 years experience a doubled risk of delivering LBW babies. Research shows that teenage mothers and women older than thirty‐5 years produce LBW infants at a rate 4.28 times higher than mothers between twenty and thirty‐4 years [8].

Numerous studies throughout Kenya reveal extensive differences in low LBW rates, which demonstrate its status as a leading public health challenge. The national data indicate that the prevalence of low birth weight is between 8% and 16%, though higher prevalence has been reported in certain regions [9, 10]. The LBW occurrence rate varies throughout different population groups and geographical locations because of maternal health conditions, economic standing, nutrition level, and prenatal care standards [11].

LBW prevalence assessment through a systematic review helps create a thorough examination of clinical data to reveal major statistical patterns, risk elements, and geographic divisions. Public health policies thrive through such reviews, which improve maternal‐child health programs and lessen the negative impacts to low‐birth‐weight incidents. The research analyzes LBW prevalence across all of Kenya and various regions and investigates two critical risk factors, which are birth order and maternal age.

## 2 Materials and Methods

### 2.1 Study Protocol

This study and its meta‐analysis findings were reported following PRISMA guidelines for systematic reviews and meta‐analyses [12]. The research process included identifying the search strategy, screening articles, evaluating study quality, collecting and extracting data, and conducting statistical analysis. Each step was carried out independently by two authors, with any discrepancies resolved through consultation with a third author.

### 2.2 Search Strategy

The literature search was conducted across multiple databases, including PubMed, CINAHL via EBSCO, Web of Science, Scopus, and Google Scholar. Searches covered all available studies up to November 16, 2024 (see Appendix X) and utilized both MeSH and non‐MeSH terms. Keywords included “incidence,” “prevalence,” “frequency,” “rate,” “epidemiology,” “occurrence,” “low birth weight,” “LBW,” “birth weight, low,” “small for gestational age,” “SGA,” “preterm,” “premature infant,” “newborn,” “neonate,” “infant,” “newly born,” and “Kenya.” The PubMed search strategy combined these terms using Boolean operators: (Incidence OR prevalence OR frequency OR rate OR epidemiology OR occurrence) AND (Low birth weight OR LBW OR birth weight, low OR small for gestational age OR SGA OR preterm OR premature infant OR newborn OR neonate OR infant OR newly born) AND (Kenya). Additionally, a manual search was performed by reviewing the reference lists of selected studies and review articles.

### 2.3 Study Selection

**Figure 1:**
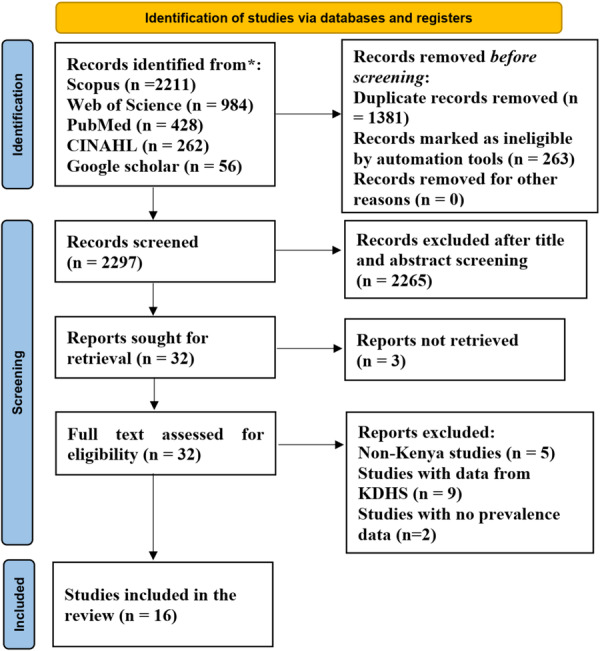
PRISMA flow chart for study selection. Where *n* (number).

Initially, all relevant articles with Kenyan‐affiliated authors were collected. After completing the search and removing duplicates, two researchers independently screened the article titles. Following this initial screening, the abstracts were reviewed. If there was any uncertainty about an article's eligibility based on the abstract, the full text was examined. In cases where the full article was unavailable, the corresponding author was contacted for access, and all steps taken to arrive at the 16 articles that were used for the systematic review were explained in Figure 1.

### 2.4 Eligibility Criteria

For this review, we selected studies that included surveys, various types of observational research, cross‐sectional analyses, or cohort (longitudinal) studies that examined LBW data (< 2500 g) alongside sociodemographic factors to evaluate the distribution of LBW across different sociodemographic groups. The primary focus was on the prevalence or proportion of LBW. Only studies utilizing survey or cross‐sectional data to assess LBW prevalence in relation to sociodemographic variables were included.

Exclusion criteria encompassed studies employing non‐random sampling methods to estimate LBW prevalence, as well as case reports, therapy‐based research, and studies on screening, diagnostic accuracy, prognosis, harm, etiology, or prevention. Additionally, letters to the editor, editorials, commentaries, reviews, and studies deemed to be of low quality were excluded.

### 2.5 Quality Assessment of Selected Article

**Figure 2:**
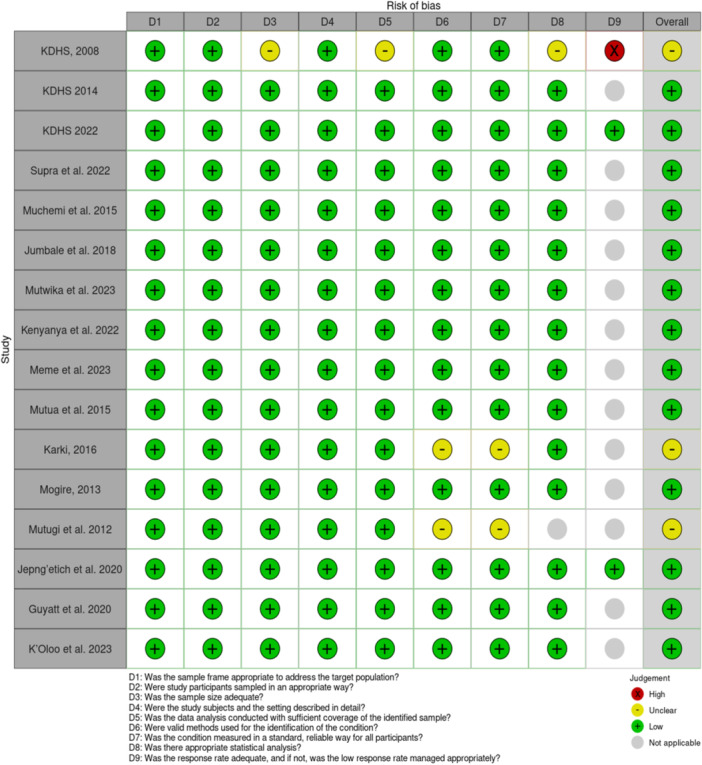
The “traffic light” plot visually represents ROB assessment, showing domain‐level judgments per outcome.

**Figure 3:**
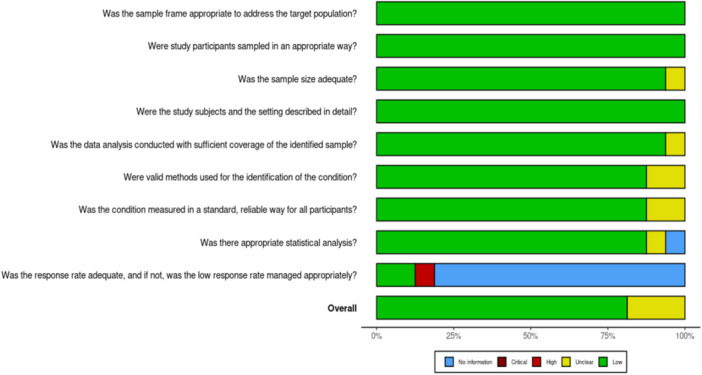
The “weighted bar” plot visually represents ROB assessment, showing domain‐level judgments per outcome.

The quality of the selected studies was evaluated using the JBI critical appraisal checklist for analytical prevalence studies [13]. This checklist consists of eight questions, with response options of “Yes,” “No,” “Unclear,” or “Not applicable.” The questions focused on assessing potential bias in each study concerning “sampling and representativeness,” “data collection and measurement,” “reliability and validity,” as well as statistical considerations and bias. Based on these criteria, studies were categorized as having a high, unclear, or low risk of bias. To ensure consistency, two researchers independently scored the articles, and any discrepancies were resolved through discussion. The RobVis app was used to generate both the traffic light plot and the weighted light plot for the risk of bias assessment (Figures 2 & 3) [14].

### 2.6 Data Extraction

A standardized form, which was initially tested on two included studies, was used to extract data on study characteristics, outcomes, and risk of bias. Two authors independently conducted the data extraction, and any discrepancies were resolved through consultation with a third reviewer acting as an arbitrator. The extracted data for each study included the study authors, publication year, total sample size, number of LBW cases, LBW prevalence, study design, state/geo‐political zone (GPZ), and residential setting (urban/rural).

### 2.7 Statistical Analysis

The prevalence of LBW in the study was determined using the total sample size and the number of LBW cases. Potential predictors of LBW were analyzed using a case‐control study design, and the prevalence event rate (PER) along with a 95% confidence interval (CI) was calculated. Study heterogeneity was assessed using the prediction interval, Cochran's Q test, and the I² statistic, with heterogeneity classified as follows: absent (0–24%), moderate (25–49%), substantial (50–75%), and considerable (> 75%) [15]. Subgroup and meta‐regression analyses were conducted to explore potential sources of heterogeneity [16]. A meta‐analysis was performed using the DerSimonian‐Laird method to apply the random effects model [17]. To evaluate regulatory impact, sensitivity analyses were carried out through stepwise elimination of specific studies to confirm validity and reliability [18]. Post‐hoc comparisons were made based on study year, study type, sample size, study quality, investigated region, and province. Publication bias was assessed using Egger's and Begg's tests [19]. All statistical analyses were conducted using Comprehensive Meta‐Analysis (CMA) software version 4, with a significance level of < 0.05 applied for all tests.

## 3 Results

The approach shown in Table 1 was merged with the elimination process for duplicate studies. After reviewing the full texts of 32 suitable publications, they identified 16 additional articles that did not qualify for inclusion based on specific requirements. The thorough assessment process confirmed quality standards for 16 eligible publications, which then advanced toward qualitative review (Table 1). The secondary data for this analysis originates from included studies as well as Demographic and Health Surveys (DHS), which ran in Kenya between 2008 and 2022/23 (USAID). The effect size index appears as a prevalence ratio that is expressed using percentages. The random‐effects model served to generalize results to equivalent studies existing in Kenya. The study's outcomes provide fundamental groundwork for researchers conducting analysis in populations with analogous ethnic and socioeconomic composition in future studies.

**Table 1:** Studies Characteristic Entered Into Meta‐analysis.

Study Name	Total Sample	Event	Prevalence	Mid‐Year	Design	Place
[20]	2728	153	5.6	2006	Cross sectional	National
[21]	6146	467	7.6	2011	Cross sectional	National
[22]	3225	274	8.5	2018	Cross sectional	National
[23]	1573	105	6.7	2019	Retrospective	Nairobi
[24]	327	40	12.3	2013	Cross sectional	Olkalou
[9]	525	145	29	2015	Cross sectional	Mvita
[10]	113	18	13.7	2018	Cross sectional	Kisumu
[25]	212	75	35.4	2019	Cross sectional	Kiambu County
[26]	323	16	5	2021	Cross sectional	Nairobi
[27]	3602	229	6.4	2010	cohort studies	Nairobi
[28]	656	31	4.7	2013	Cross sectional	National
[29]	405	133	32.8	2011	Cross sectional	Nairobi
[30]	327	57	16.4	2011	Cross sectional	Narok
[31]	178	27	16.6	2019	Cross sectional	Kapsabet
[15]	1004	90	9	2019	Cross sectional	Machakos County
[32]	9139	1151	12.6	2020	Cross sectional	National

### 3.1 Pooled Prevalence of Low Birth Weight

**Figure 4:**
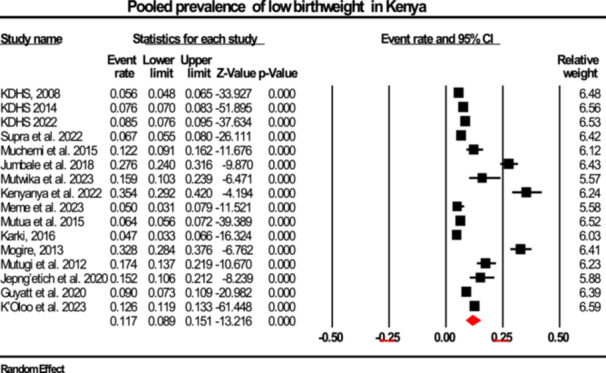
Forest plot illustrating the prevalence of low birth weight in Kenya, presented with 95% confidence intervals (CI), the event rate, and relative weight.

The primary analysis estimated the pooled prevalence of LBW across studies conducted in Kenya. The pooled prevalence was 11.7% (95% CI: 8.9%–15.1%), with substantial heterogeneity between studies (Q = 766.8, df = 15, *p* < 0.001; I² = 98%). This indicates considerable variation in LBW prevalence estimates beyond chance, supporting the use of a random‐effects model. A 95% prediction interval suggests the true prevalence in similar settings could range broadly, highlighting the importance of investigating sources of heterogeneity (Figure 4).

### 3.2 Sub‐Group Analysis

Subgroup analyses were performed to identify possible heterogeneity contributors of low birth weight (LBW) prevalence estimations. In all assessed subgroups, i.e., wealthy quintile, location of residence, maternal age, birth order, and maternal education, a very high amount of between‐study heterogeneity occurred, indicating considerable variability beyond random error.

Although no statistically significant differences were observed in LBW prevalence between the wealth quintiles, residency levels, and maternal age groups (all *p* > 0.05), the subgroup analysis showed that there was statistically significant heterogeneity as regards to the maternal age group (*p* = 0.002), and residential status (*p* = 0.0030) (Table 2).

**Table 2:** Subgroup Analysis of LBW Based on Region, Wealth Quantile, Residence, Mother's Education, Birth Order, and Maternal Age.

Variable	Sub‐group	Studies No.	Sample (*N*)		Pooled prev. (%)	Heterogeneity		
			All	Event		Pred. Interval	I^2^	*p*‐value
Region	Central	5	1397	230	11.9	(0.026, 0.407)	96.046	< 0.001
	Coast	4	1857	257	11.7	(0.025, 0.408)	97.162	< 0.001
	Eastern	4	2583	230	8.80	(0.018, 0.334)	0.000	0.959
	Nairobi	7	7321	626	9.40	(0.021, 0.334)	97.902	< 0.001
	North Eastern	3	966	87	9.20	(0.017, 0.370)	0.000	0.556
	Nyanza	3	2426	144	5.50	(0.010, 0.242)	77.670	< 0.001
	Rift valley	5	2569	222	8.90	(0.019, 0.334)	93.515	< 0.001
	Western	4	1568	84	6.70	(0.014, 0.276)	89.420	< 0.001
	Overall	35	23213	1880	9.00	(0.023, 0.292)	95.470	< 0.001
	Test for subgroup differences: Tau‐TauSq = 0.722–0.521, Q = 0.745, df (Q) = 7, *p*‐value = 0.404							
Wealth Quantile	Lowest	3	1622	127	7.40	(0.037, 0.141)	80.953	0.005
	Second	3	2053	160	8.20	(0.042, 0.154)	84.999	0.001
	Middle	3	2225	174	7.70	(0.039, 0.145)	0.000	0.396
	Fourth	3	2798	222	7.40	(0.038, 0.139)	84.955	0.001
	Highest	3	3401	219	6.00	(0.031, 0.115)	84.955	0.020
	Overall	15	12099	902	7.30	(0.044, 0.118)	74.537	< 0.001
	Test for subgroup differences: Tau‐TauSq = 0.274–0.075, Q = 2.002, df (Q) = 4, *p*‐value = 0.735							
Residence	Rural	8	9023	751		(0.030, 0.319)	92.147	< 0.001
	Urban	11	21460	2268		(0.031, 0.317)	98.422	< 0.001
	Overall	19	30483	3019		(0.033, 0.303)	97.671	< 0.001
	Test for subgroup differences: Tau‐TauSq = 0.607–0.368, Q = 0.003, df (Q) = 1, *p*‐value = 0.955							
Mother's Education	No Education	5	1617	198		(0.032, 0.411)	96.242	< 0.001
	Primary	5	4104	404		(0.029, 0.385)	97.079	< 0.001
	Secondary	5	4368	353		(0.022, 0.320)	92.221	< 0.001
	Tertiary	5	4634	323		(0.020, 0.299)	93.644	< 0.001
	Overall	20	14723	1278		(0.030, 0.313)	95.458	< 0.001
	Test for subgroup differences: Tau‐TauSq = 0.656–0.430, Q = 6.719, df (Q) = 3, *p*‐value = 0.033							
Birth order	1st order	4	4250	376		(0.059, 0.025)	79.441	0.002
	2nd & 3rd order	4	6116	401		(0.044, 0.094)	48.693	0.119
	4th & 5th order	3	2083	124		(0.038, 0.090)	17.923	0.296
	6th and above	3	1168	95		(0.052, 0.123)	16.909	0.300
	Overall	14	13617	996		(0.045, 0.114)	75.731	< 0.001
	Test for subgroup differences: Tau‐TauSq = 0.158–0.025, Q = 8.467, df (Q) = 3, *p*‐value = 0.037							
Maternity Age	< 20	4	2211	194		(0.057, 0.133)	65.740	0.033
	20–34	4	9347	656		(0.047, 0.106)	79.126	0.002
	35–49	4	1544	138		(0.057, 0.136)	18.163	0.300
	Overall	12	13102	988		(0.052, 0.122)	72.692	< 0.0001
	Test for subgroup differences: Tau‐TauSq = 0.172–0.030, Q = 3.269, df (Q) = 2, *p*‐value = 0.195							

#### 3.2.1 Prevalence By Mother's Age

**Figure 5:**
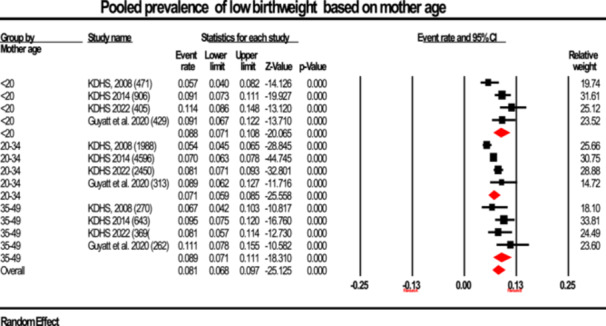
Forest plot illustrating the prevalence of low birth weight stratified according to mother's age, presented with 95% confidence intervals (CI), the event rate, and relative weight.

We evaluated the relationship between maternal age and the prevalence of low birth weight (LBW). The pooled prevalence of LBW was 8.8% (95% CI: 7.1%–10.8%; I² = 65.7%) among mothers younger than 20 years, 7.1% (95% CI: 5.9%–8.5%; I² = 79.1%) among those aged 20–34 years, and 8.9% (95% CI: 6.8%–9.7%; I² = 18.2%) among mothers aged 35–49 years. The interaction between age and age groups could not show a statistically significant variance (Q = 3.27, df = 2, *p* = 0.20). The differences in LBW prevalence across maternal age subgroups were not statistically significant (Tau² = 0.172–0.030, Q = 3.269, df(Q) = 2, *p* = 0.195) (Figure 5).

#### 3.2.2 Prevalence By Residence

**Figure 6:**
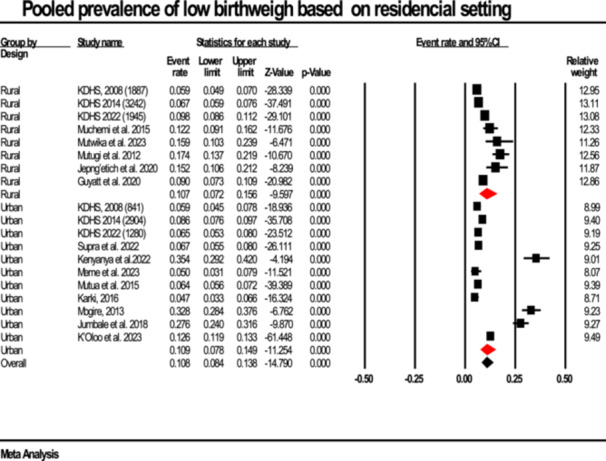
Forest plot illustrating the prevalence of low birth weight stratified according to ressidence, presented with 95% confidence intervals (CI), the event rate, and relative weight.

The analysis assessed whether residence (urban vs rural) was associated with LBW prevalence. The pooled estimates were similar, each at 10.7% (rural 95% CI: 7.2%–15.6%; urban 95% CI: 7.8%–14.7%). There was no evidence of a statistically significant difference between residential settings (Q = 3.75, df = 7, *p* = 0.81). Substantial heterogeneity persisted (I²: 92%–98%) (Figure 6).

#### 3.2.3 Prevalence By Region

**Figure 7:**
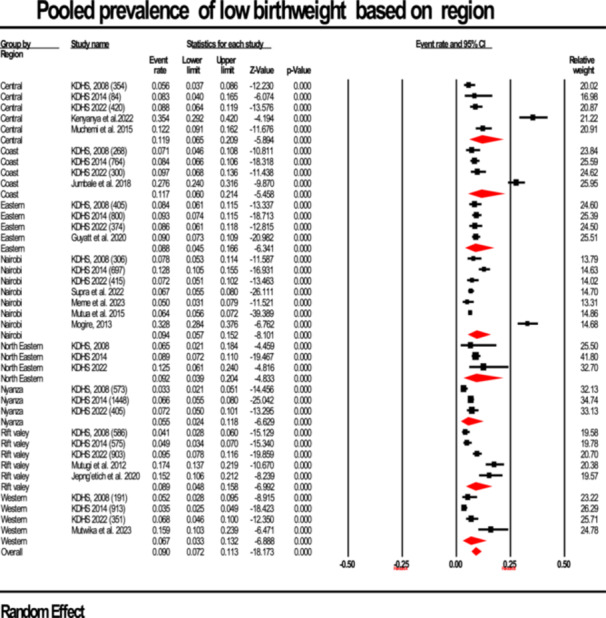
Forest plot illustrating the prevalence of low birth weight stratified according to region, presented with 95% confidence intervals (CI), the event rate, and relative weight.

The prevalence of LBW by region was estimated, whereby differences were not statistically significant (Q = 0.75, df = 7, *p* = 0.16). Regions with the highest pooled estimates were Central (11.9%; 95% CI: 6.5%–20.9%; I^2^: 96.0%) and Coast (11.7%; 95% CI: 6.0%–21.4%; I^2^: 97.2%) regions (6.7%; 95% CI: 6.5%–20.9%; I²: 89.42%). In contrast, the lowest LBW prevalence was recorded in the Nyanza region at 5.5% (95% CI: 6.5%–20.9%; I²: 77.67%). The amount of heterogeneity was high in all regions as shown in Figure 7.

#### 3.2.4 Prevalence By Wealth Quantile

**Figure 8:**
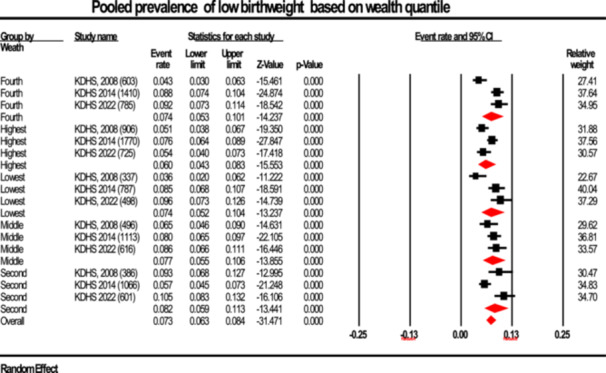
Forest plot illustrating the prevalence of low birth weight stratified according to wealth quantile, presented with 95% confidence intervals (CI), the event rate, and relative weight.

Figure 8 presents the pooled prevalence estimates of LBW across different wealth quintiles. The estimated prevalence rates for the lowest, second, middle, fourth, and highest wealth quintiles were 7.4% (95% CI: 7.6%–21.8%; I²: 80.95%), 8.2% (95% CI: 7.1%–19.9%; I²: 84.99%), 7.7% (95% CI: 5.4%–15.7%; I²: 0.0%), 7.4% (95% CI: 4.7%–7.82%; I²: 84.95%), and 6.8% (95% CI: 5.0%–9.1%; I²: 84.95%), respectively. The analysis did not reveal any statistically significant differences or discernible patterns in LBW prevalence across wealth quintiles (Test for subgroup differences: Tau² = 0.274–0.075, Q = 2.00, df(Q) = 4, *p* = 0.74).

#### 3.2.5 Prevalence By Birth Order

**Figure 9:**
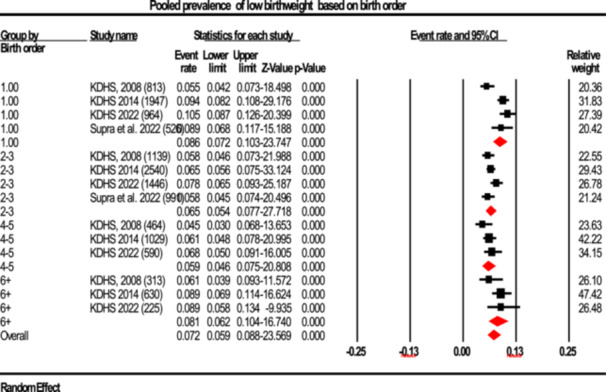
Forest plot illustrating the prevalence of low birth weight stratified according to birth order, presented with 95% confidence intervals (CI), the event rate, and relative weight.

The pooled prevalence estimates of LBW based on birth order were 8.6% for first‐borns (95% CI: 7.2%–10.3%; I²: 79.44%), 6.5% for second and third births (95% CI: 5.4%–7.7%; I²: 48.69%), 5.9% for fourth and fifth births (95% CI: 4.6%–7.5%; I²: 17.92%), and 8.1% for sixth or higher‐order births (95% CI: 6.2%–10.4%; I²: 16.91%). Notably, LBW prevalence was significantly lower among fourth and fifth births compared to first‐borns and those of birth order six or higher (Figure 9).

#### 3.2.6 Prevalence By Mother's Education

**Figure 10:**
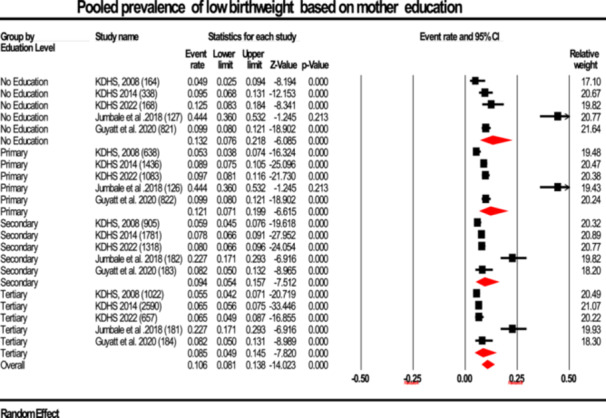
Forest plot illustrating the prevalence of low birth weight stratified according to mother's education, presented with 95% confidence intervals (CI), the event rate, and relative weight.

To evaluate whether maternal education level influences LBW prevalence, subgroup analyses were performed. Mothers with no formal education had the highest pooled prevalence at 13.2% (95% CI: 8.9%–11.4%), while mothers with tertiary education showed the lowest at 8.5% (95% CI: 5.8%–7.9%). Differences between education levels were statistically significant (Q = 6.72, df = 3, *p* = 0.03). Heterogeneity within subgroups remained high (I²: 92%–97%), indicating other factors may contribute to variation (Figure 10).

### 3.3 Meta‐Regression By Year of Study

**Figure 11:**
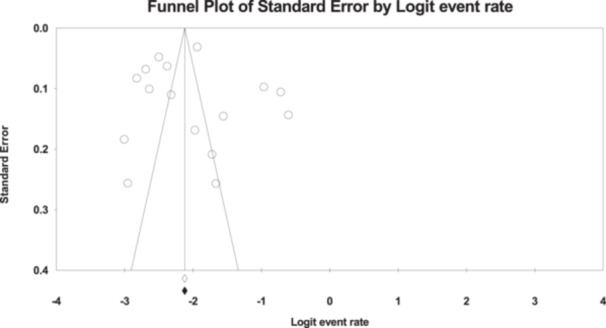
Funnel Plot Assessing Publication Bias.

A meta‐regression examined whether LBW prevalence changed over time. Although the prevalence declined slightly with more recent publication years, the trend was not statistically significant (Q = 0.68, df = 1, *p* = 0.41). Significant heterogeneity persisted (Q = 2940.54; I²: 98.5%), suggesting that factors other than publication year may explain the variability across studies (Figure 11).

## 4 Discussion

The pooled prevalence of LBW in Kenya was estimated at 11.7% (95% CI: 8.9%–15.1%), aligning with the persistent public health concern reported across many LMICs in Sub‐Saharan Africa and South Asia [2, 11]. This finding underscores that despite national health programs, LBW remains a substantial burden in Kenya, comparable to Ethiopia (13%) (33), Nigeria (12–14%) (3), and India (15–20%) [7, 33]. LBW research demonstrates substantial variations in its prevalence, which demonstrates the unique nature of this health problem, yet declares a need for interrelated strategies to resolve its root causes [34]. Despite a global commitment to reduce LBW by 30% by 2025 [35], this study underscores that LBW remains a major public health burden in Kenya, aligning with recent global estimates that place Sub‐Saharan Africa among the regions with the highest LBW prevalence [2, 11].

Although mothers below 20 and above 35 years showed higher LBW prevalence (8.8% and 8.1% respectively) than those aged 20–34 years (7.1%), these differences were not statistically significant (*p* = 0.195) (Table 2; Figure 5). This is somewhat unexpected, given established evidence that from researchers in Indonesia [36] and India [37] where LBW has been associated with adolescent pregnancies and old maternal age because of biological insufficiency or complications of gestation e.g., preeclampsia and gestational diabetes [8, 36]. Scientific evidence shows mothers from ages 20 below and 35 above tend to deliver infants with LBW as a result of multiple biological and socioeconomic elements [36]. The condition of being older and pregnant increases the possibility of developing preeclampsia and gestational diabetes, which eventually leads to LBW [38]. However, the lack of any significant variation in this meta‐analysis suggests that, although these age brackets are conventionally considered to be high risk, a more extensive or more widely‐grained set of data is required in order to assert that age is a proven variable risk factor in Kenya.

There were observed regional differences with the highest prevalence of LBW in the Central and Coast regions (11.9% and 11.7%) and lower rates in Nyanza and Western (5.5% and 6.7%). Nonetheless, these differences were not significant in the subgroup analysis thereof (*p* = 0.159) as shown in Table 2; Figure 7. This finding resonates with research in Ethiopia, where regional heterogeneity of LBW was attributed more to socioeconomic disparities than geography alone [39, 40]. This suggests that broader systemic factors, such as healthcare access, quality of antenatal care, and maternal nutrition, may be more influential than residential status alone as observed in studies done in Nigerian and Ethiopian [34, 41].

However, there was no significant association between wealth and LBW prevalence (*p* = 0.735; Table 2; Figure 8). This finding can be compared to recent publications in Kenya and India, indicating that wealth alone might not be enough to protect against LBW without better maternal education, access to healthcare, as well as diet diversity [41, 42].

Results indicated that the connection between birth order and LBW prevalence was statistically significant (*p* = 0.037). Higher prevalence among first‐born (8.6%) as well as sixth‐or‐higher‐order births (8.1%) was observed compared to fourth‐ and fifth‐order births (5.9%) (Table 2; Figure 9). The trend is in line with results in West Bengal, India, where first pregnancies had greater odds of LBW owing to physiological immaturity and inexperience in handling pregnancy, leading to decreases in observed outcomes [37]. In the case of higher‐ordered births, depletion syndrome in the mother may be a factor; repeated pregnancies mean a lack of time to recover maternal stores, further deprived of nutrients during fetal development [43]. This highlights the need and relevance of most appropriate interventions like family planning and nutrition among the high‐parity women.

Maternal education was confirmed in this review as the main determinant of LBW in Kenya. After making such comparisons, the prevalence of LBW was the highest in mothers who did not have any formal education (13.2%) and was the lowest in mothers with tertiary education (8.5%; *p* = 0.03; Table 2; Figure 10). These results coincide with solid evidence of other LMICs that maternal education is strongly linked to better health‐seeking behavior, nutrition, and antenatal care, all of which diminish the risks of a low birth weight [44, 45]. The extent of education increases the maternal awareness and ability to make good use of healthcare services, a circumstance that has been found to be effective greatly on birth outcomes in diverse settings [38].

### 4.1 Strengths and Limitations

The major strength of this review is the rigorous methodology: following the PRISMA framework, there has been the implementation of multiple databases and subgroup and meta‐regression analyses were rigorously performed. These significant I^2^ values (even 98%) show a high heterogeneity due to a wide variety of contexts and methodological differences of the various included studies. This must be read with caution, although it also reflects the benefits of random‐effects modeling in generalizing results of research to analogous settings. This study has several limitations. Most of the included studies were cross‐sectional in design, which limits causal inference. In addition, key covariates associated with low birth weight—such as maternal nutritional status and maternal infections—were not consistently reported and therefore could not be accounted for in the analysis.

## 5 Conclusion

This meta‐analysis establishes that LBW is a relevant issue of public health in Kenya with a combined prevalence of 11.7% (95% CI: 8.9–15.1) and high heterogeneity. The order of birth and the education of the mother also proved to be a major determinant factor, whereby LBW was highest among the mothers who had not had any formal education and those with 1st or high‐parity children, and lowest among the mothers who had had a tertiary education and those with 4th‐5th‐order children. On the contrary, maternal age, residence, wealth, and region appeared to have no pattern effect. To minimize LBW, the focus must be dedicated to female education and those aspects of potential family planning to cover the threats of high‐parity births, as well as on equitable delivery of quality ANC care to all regions and socioeconomic groups. Future studies are required to consider longitudinal methods to get more insight into the interaction of biological and environmental discriminating factors that affect LBW.

## Author Contributions

**Wusa Makena:** conceptualization, methodology, formal analysis, investigation, writing – review and editing. **Monday Nwankwo:** conceptualization, methodology, formal analysis, investigation, writing – review and editing. **Aisha Idris:** data curation, investigation, resources, software, writing – review and editing. **Onyinoyi Bethel Onimisi:** data curation, investigation, resources, software, writing – review and editing. **Moses Sunday Adefisayo:** investigation, validation, writing – review and editing. **Patrick Maduabuchi Aja:** investigation, validation, writing – review and editing. **Ibe Michael Usman:** data curation, investigation, validation, formal analysis, writing – review and editing. **Elna Owembabazi:** data curation, investigation, validation, formal analysis, writing – review and editing. **Elizabeth Bassy Umoren:** supervision, validation, visualization, writing – original draft and editing. **Ilemobayo Victor Fasogbon:** writing – original draft, formal analysis, writing – review and editing. **Onyinye Vivian Ojiakor:** writing – original draft, formal analysis, writing – review and editing. **Chinyere Nkemjika Anyanwu:** writing – original draft, formal analysis, writing – review and editing.

## Funding

The authors have nothing to report.

## Conflicts of Interest

The authors declare no conflicts of interest.

## Transparency Statement

The lead author Wusa Makena affirms that this manuscript is an honest, accurate, and transparent account of the study being reported; that no important aspects of the study have been omitted; and that any discrepancies from the study as planned (and, if relevant, registered) have been explained.

## Supporting information

Supporting File

## Data Availability

Data sharing is not applicable to this article, as no new datasets were generated during the current study. Quantitative data were extracted from published primary studies included in the meta‐analysis, and all source data are available in the respective original publications.
