# Supplementary material for: Disparities in Low‐Birth‐Weight Prevalence Across Kenya: A Systematic Review and Meta‐Analysis of Maternal, Demographic, and Socioeconomic Risk Factors
Source: Health Sci Rep. 2026 Apr 19;9(4):e72404. doi: 10.1002/hsr2.72404 (PMC13092218; doi:10.1002/hsr2.72404)
Supplement: Supplementary file 1 — Supporting File [file HSR2-9-e72404-s001.docx]

**PubMed (MEDLINE) Search Strategy**

(Incidence[Title/Abstract] OR prevalence[Title/Abstract] OR frequency[Title/Abstract] OR rate[Title/Abstract] OR epidemiology[Title/Abstract] OR occurrence[Title/Abstract]) AND ("Low Birth Weight"[Mesh] OR "low birth weight"[Title/Abstract] OR LBW[Title/Abstract] OR "birth weight, low"[Title/Abstract] OR "small for gestational age"[Title/Abstract] OR SGA[Title/Abstract] OR preterm[Title/Abstract] OR "premature infant"[Title/Abstract]) AND (newborn[Title/Abstract] OR neonate[Title/Abstract] OR infant[Title/Abstract] OR "newly born"[Title/Abstract]) AND (Kenya[Title/Abstract])

Filters: Humans, English, Publication date from 2000/01/01 to 2024/11/16

**Scopus Search Strategy**

TITLE-ABS-KEY ("low birth weight" OR LBW OR "birth weight, low" OR "small for gestational age" OR SGA OR preterm OR "premature infant") AND TITLE-ABS-KEY (incidence OR prevalence OR frequency OR rate OR epidemiology OR occurrence) AND TITLE-ABS-KEY (newborn OR neonate OR infant OR "newly born") AND TITLE-ABS-KEY (Kenya) AND (LIMIT-TO (LANGUAGE, "English")) AND (PUBYEAR > 1999)

**Web of Science Search Strategy**

TS = ("low birth weight" OR LBW OR "birth weight, low" OR "small for gestational age" OR SGA OR preterm OR "premature infant") AND TS = (incidence OR prevalence OR frequency OR rate OR epidemiology OR occurrence) AND TS = (newborn OR neonate OR infant OR "newly born") AND TS = (Kenya)

Refined by:

- Language: English

- Document Types: Article

- Timespan: 2000–2024

**CINAHL (EBSCOhost) Search Strategy**

(MH "Infant, Low Birth Weight" OR "low birth weight" OR LBW OR "small for gestational age" OR SGA OR preterm OR "premature infant") AND (MH "Prevalence" OR incidence OR prevalence OR frequency OR rate OR epidemiology OR occurrence) AND (newborn OR neonate OR infant OR "newly born") AND (Kenya)

Limiters:

- English Language

- Human

- Published Date: 2000–2024
